# The Influence of Clinical and Patient-Reported Outcomes on Post-surgery Satisfaction in Cholecystectomy Patients

**DOI:** 10.1007/s00268-017-3917-7

**Published:** 2017-03-09

**Authors:** K. A. McLean, Z. Sheng, S. O’Neill, K. Boyce, C. Jones, S. J. Wigmore, E. M. Harrison

**Affiliations:** Clinical Surgery, University of Edinburgh, Royal Infirmary of Edinburgh, 51 Little France Crescent, Edinburgh, EH16 4SA Scotland

## Abstract

**Background:**

Patient-reported outcomes (PROMs) and post-operative satisfaction have become a growing focus of surgical outcome evaluation and are considered key components of the movement towards patient-centred care. The aim was to compare the association of traditional clinical outcome measures and PROMs with post-surgery satisfaction in cholecystectomy patients.

**Methods:**

Patients who had undergone elective or emergency cholecystectomy for gallstone disease were sent validated PROM questionnaires, and telephone follow-up was performed in all cases. Categorical data were compared with the Chi-square and Fisher’s exact tests. Satisfaction was investigated using a “top-box” approach, and multivariable logistic regression was performed for factors significantly (*p* < 0.05) associated with satisfaction in univariable analyses.

**Results:**

A total of 234 patients underwent cholecystectomy between 1 March 2014 and 1 May 2014, and 147 patients (63%) completed the questionnaire. 104/147 (71%) reported being “very satisfied” with their surgical outcome. In univariable analyses, satisfaction showed significant association with an absence of hospital-recorded 30-day complications (OR = 4.11, 95% CI 1.29–13.84), but not re-attendance, readmission, or length of stay. In a multiple regression analysis, no traditional clinical outcome measures were associated with satisfaction. By contrast, self-perceived health (OR = 4.04, 95% CI 1.44–11.86), the absence of patient-reported wound pain (OR = 6.11, 95% CI 1.83–21.74), and a return to normal leisure activities (OR = 11.14, 95% CI 2.61–55.26) were associated with satisfaction.

**Conclusion:**

PROMs are the major determinants of patient satisfaction following cholecystectomy. When assessing outcomes following cholecystectomy, the measurement of clinical outcomes alone is inadequate and should be supplemented by the use of PROMs.

## Introduction

Biliary pain secondary to cholelithiasis is among the most common gastrointestinal causes of hospitalisation [1, 2], and laparoscopic cholecystectomy is considered the “gold standard” treatment for symptomatic cholelithiasis [3].

Patient-reported outcomes measures (PROMs), including satisfaction and health-related quality of life (HRQoL), have been increasingly recognised as potentially invaluable tools in both the assessment and quality improvement in healthcare interventions [4]. The isolated use of traditional, objective clinical outcomes following surgery can fail to recognise factors which are important to patients, and the contribution the perspective of patients can have in healthcare appraisal. It has been said that the ultimate measure by which to judge the quality of a medical effort is whether it helps patients as they see it [5]. As such, PROMs have become a growing focus of surgical outcome evaluation [4, 6] and are considered key to the movement towards more patient-centred healthcare [7]. These also have the potential to expose the hidden burden of surgical interventions which may otherwise be self-managed or addressed in primary care.

This study aimed to determine the relationship of clinical outcome measures and PROMs with post-surgery satisfaction in a cohort of cholecystectomy patients within a 3-month post-operative period.

## Materials and methods

### Patients/recruitment

Consecutive patients were recruited prospectively and underwent cholecystectomy (open or laparoscopic) at the Royal Infirmary of Edinburgh from the 1 March 2014 to the 1 May 2014. This included all patients over 18 years and excluded cholecystectomies due to known gallbladder cancer or resulting from another surgical procedure.

### Data collection

The electronic patient record was used to obtain sociodemographic, clinical, and contact information. PROMs were assessed using combined general (EQ-5D-5L [8]) and condition-specific [9, 10] questionnaires (Appendix 1) and focussed on post-operative symptoms, post-operative function, and satisfaction. A questionnaire was sent via post to the home address of each patient and was supplemented by a telephone interview. The follow-up interviews were performed by independent individuals trained for the purposes of the study (KAM, ZS) and had no knowledge of the details of surgery or recovery. These were completed between 1- and 3-month post-cholecystectomy, and three separate attempts were made to contact each patient by telephone. Institutional ethical approval was obtained and informed consent gained from each patient contacted (Appendix 2).

### Statistical analyses

The questionnaire responses were assessed using either a binary or five-point Likert scale and combined into dichotomous categories for analytic purposes. The patient satisfaction variable was investigated using a “top-box” approach comparing those who were “*very satisfied*” (5) to those who were not *“very satisfied*” (1–4), as previously described [11–13]. All other Likert variables were aggregated into “*low*” (1–2), and “*high*” (3–5) responses. In addition, we summarised the association between paired hospital-recorded and patient-reported 30-day complication rates using a McNemar odds ratio.

Continuous data were summarised as a mean and range and compared using *t* tests. Categorical data were cross-tabulated and differences in proportions tested using the Chi-square or Fisher’s exact test. Where appropriate, an odds ratio (OR) and 95% confidence interval (95% CI) were calculated. Multivariable logistic regression was performed with variables identified as significantly associated with satisfaction on univariable analysis. R Studio v2.1 (R Foundation for Statistical Computing) was used for statistical analyses, and *p* < 0.05 was considered to be statistically significant.

## Results

A total of 234 consecutive patients were recruited during the study period. Of these, 165 (71%) responded to the questionnaire by either post or telephone; however, 18 (8%) questionnaires were returned incomplete and so were excluded. Six (3%) refused participation, 63 (27%) did not respond, and so 147 (63%) were included in the analyses. The characteristics of respondent and non-respondents were similar (Table 1) with respondents being older than non-respondents (57.7 years vs. 48.5 years, *p* < 0.001) and with a higher ASA classification (*p* = 0.044). One hundred and four respondents (71%) reported being “*very satisfied*” with the outcome of their cholecystectomy, 37 (25%) being “*satisfied*” (an overall satisfaction rate of 96%), and 6 (4%) reporting feeling “*worse*”, “*no effect*”, or “*do not know*”. No baseline differences were seen between satisfaction groups (Table 2), including the operative approach utilised, type of admission (acute, delayed, or elective), or time post-operative.

**Table 1:** Demographic and pre-operative characteristics of respondents and non-respondents

		Patient-reported satisfaction		
		Respondents *n* = 147	Non-respondents *n* = 87	*p* value
Demographics				
Age (years)				
Mean (range)		57 (22–86)	49 (20–84)	<0.001
BMI (kg/m^2^)				
Mean (range)		30 (18–48)	29 (17–42)	0.380
Gender				
Male		44 (30)	17 (19)	0.091
Female		103 (70)	70 (81)	
Pre-operative characteristics				
Type of admission				
Acute		52 (35)	28 (32)	0.469
Elective		29 (20)	13 (15)	
Delayed		66 (45)	46 (53)	
ASA				
ASA 1		57 (39)	45 (52)	0.044
ASA 2		69 (47)	35 (40)	
ASA ≥ 3		20 (14)	7 (8)	
Indication				
Biliary colic		75 (51)	41 (47)	0.440
Cholecystitis		42 (29)	28 (32)	
Gallstone pancreatitis		11 (8)	11 (13)	
CBD stones		16 (11)	7 (8)	
Other ^a^		3 (1)	0 (0)	
Approach				
Laparoscopic		132 (90)	83 (95)	0.346
Open		2 (1)	0 (0)	
Laparoscopic to open		13 (9)	4 (5)	

Data are *n* (%) unless otherwise stated

^a^Polyps (*n* = 2) and Acalculous (*n* = 1)

**Table 2:** Demographic and pre-operative characteristics of the satisfaction subgroups

		Patient-reported satisfaction		
		Not very satisfied *n* = 43	Very satisfied *n* = 104	*p* value
Demographics				
Age (years)				
Mean (range)		56 (24–85)	57 (22–86)	0.603
BMI (kg/m^2^)				
Mean (range)		30 (21–46)	30 (18–48)	0.580
Gender				
Male		13 (30)	31 (30)	1.000
Female		30 (70)	73 (70)	
Pre-operative characteristics				
Type of admission				
Acute		15 (35)	51 (49)	0.255
Elective		19 (44)	33 (32)	
Delayed		9 (21)	20 (19)	
ASA ^a^				
ASA 1		18 (43)	39 (38)	0.881
ASA 2		18 (43)	51 (49)	
ASA ≥ 3		6 (14)	14 (13)	
Indication				
Biliary colic		23 (54)	52 (50)	0.964
Cholecystitis		12 (28)	30 (29)	
Gallstone pancreatitis		2 (5)	9 (9)	
CBD stones		4 (9)	12 (12)	
Other ^b^		2 (5)	1 (1)	
Approach				
Laparoscopic		36 (84)	96 (92)	0.095
Open		0 (0)	2 (2)	
Laparoscopic to open		7 (16)	6 (6)	
Time post-operative				
≤30 days		12 (28)	29 (28)	0.971
31–60 days		20 (47)	51 (49)	
>60 days		11 (26)	24 (23)	

Data are *n* (%) unless otherwise stated

^a^ Not very satisfied *n* = 42

^b^ Polyps (*n* = 2) and Acalculous (*n* = 1)

When the associations between post-operative satisfaction and post-operative symptoms were explored (Table 3), the absence of patient-reported 30-day complications such as wound pain [OR: 5.98 (2.39–15.60), *p* < 0.001], or other symptoms [OR: 2.42 (1.04–5.63), *p* = 0.026] showed significant association with high satisfaction. Furthermore, the absence of recurrent symptoms [OR: 5.08 (1.76–15.61), *p* = 0.001], or de novo heartburn [OR: 3.29 (1.24–8.86), *p* = 0.014] was also significantly associated with satisfaction.

**Table 3:** Univariate association of PROM and clinical outcomes with patient-reported satisfaction, and multivariate logistic regression of significant variables

			Patient-reported satisfaction					
			Not very satisfied *n* = 43	Very satisfied *n* = 104	Univariate association		Multivariate logistic regression	
					OR (95% CI)	*p* value	OR (95% CI)	*p* value
Post-operative symptoms								
30-day period								
No complications			27 (63)	78 (75)	1.77 (0.77–4.05)	0.161	–	–
No wound infection			37 (86)	97 (93)	2.23 (0.58–8.34)	0.202	–	–
No fever			37 (86)	98 (94)	2.63 (0.66–10.52)	0.110	–	–
No wound pain			24 (56)	92 (88)	**5.98 (2.39–15.60)**	**<0.001**	**7.32 (2.35–24.46)**	**<0.001**
No other symptoms			26 (60)	82 (79)	**2.42 (1.04–5.63)**	**0.026**	1.23 (0.38–3.79)	0.721
Overall period								
No recurrent symptoms			30 (70)	95 (91)	**5.08 (1.76–15.61)**	**0.001**	1.96 (0.45–8.40)	0.361
No de novo heartburn			30 (70)	92 (88)	**3.29 (1.24–8.86)**	**0.014**	1.26 (0.37–4.12)	0.704
Post-operative function								
Minimal issues in prior 4 weeks with ^a^:								
Fatigue			19 (44)	72 (69)	**2.91 (1.32–6.53)**	**0.005**	1.07 (0.37–2.90)	0.903
Daily duties			27 (63)	93 (89)	**5.10 (1.90–14.30)**	**<0.001**	0.38 (0.07–1.91)	0.253
Leisure activities			26 (60)	97 (93)	**10.35 (3.47–35.46)**	**<0.001**	**12.19 (2.87–59.83)**	**0.001**
Maintaining relationships			40 (93)	99 (95)	1.64 (0.13–14.91)	0.629	–	–
Changes in mood			30 (70)	92 (88)	**3.59 (1.33–9.90)**	**0.006**	1.13 (0.25–4.68)	0.867
Minimal issues at present with ^a^:								
Mobility			36 (84)	89 (86)	1.23 (0.39–3.60)	0.796	–	–
Self-care			41 (95)	101 (97)	2.45 (0.17–34.83)	0.581	–	–
Usual activities			32 (74)	96 (92)	**4.65 (1.50–15.46)**	**0.004**	0.75 (0.12 –4.62)	0.749
Pain/discomfort			34 (79)	95 (91)	**3.56 (1.08–12.20)**	**0.020**	0.75 (0.10–4.76)	0.765
Anxiety/depression			34 (79)	93 (89)	2.18 (0.68–6.71)	0.164	–	–
Self-perception								
High opinion of health ^b^			12 (28)	69 (66)	**5.16 (2.24–12.58)**	**<0.001**	**3.87 (1.39–11.23)**	**0.010**
Clinical outcomes								
30-day outcomes								
No hospital-recorded 30-day complications			33 (77)	96 (92)	**4.11 (1.29–13.84)**	**0.009**	4.31 (0.93–20.81)	0.063
No A&E re-attendance			37 (86)	96 (92)	1.94 (0.52–6.87)	0.353	–	–
No readmission			42 (98)	101 (97)	0.80 (0.01–10.33)	1.000	–	–
Length of stay								
≤1d			22 (51)	43 (41)	–	0.632	–	–
2–7d			14 (33)	48 (46)				
>7d			7 (16)	13 (13)				

Statistically significant values (*p* < 0.05) are given in bold

Data are *n* (%) unless otherwise stated

^a^ Response of “no problems”/“slight problems”

^b^ Numerical rating score ≥8/10

The association of patient-reported physical, emotional, and social functionality with patient satisfaction was explored. Satisfaction was strongly associated with a quick return to daily duties [ORs: 5.10 (1.90–14.30), *p* < 0.001] and leisure activities [10.35 (3.47–35.46), *p* < 0.001], together with minimal post-operative fatigue [OR: 2.91 (1.32–6.53), *p* = 0.005] or mood alterations [OR: 3.59 (1.33–9.90), *p* = 0.006]. Similarly, when evaluating current health, satisfaction was associated with the absence of pain [OR: 3.56 (1.08–12.20), *p* = 0.020] and high self-perceived health [OR: 5.16 (2.24–12.58), *p* < 0.001].

In an analysis of traditional clinical outcome measures, satisfaction was associated with the absence of hospital-recorded 30-day complications [OR: 4.11 (1.29–13.84), *p* = 0.009], but not re-attendance, readmission, nor length of stay.

Multivariable logistic regression revealed that the absence of patient-reported wound pain [OR: 7.32 (2.35–24.46), *p* < 0.001], a return to normal leisure activities [OR: 12.19 (2.87–59.83), *p* = 0.001], and a high self-perceived opinion of current health [OR: 3.87 (1.39–11.23), *p* = 0.010] were independently associated with higher post-cholecystectomy satisfaction.

Finally, a McNemar odds ratio (mOR) was used to summarise the association in paired patient-reported and hospital-recorded 30-day complication rates (Table 4). Where the patient-reported and hospital-recorded 30-day complication rates differed for a patient, it was 5.17 times more likely [95% CI (2.12–15.15), *p* < 0.001] that patient-reported complications were present (without a hospital record) than hospital-recorded complications (without being reported by the patient).

**Table 4:** McNemar odds ratio for paired hospital-recorded and patient-reported 30-day complication rates (n = 146)

	Hospital-recorded 30-day complications		McNemar OR (95% CI)	*p* value
	Absent	Present	5.17 (2.12–15.15)	<0.001
Patient-reported 30-day complications				
Absent	98	6		
Present	31	11		

## Discussion

The purpose of this study was to determine the relationship of clinical outcome measures and PROMs with post-surgery satisfaction in a cohort of cholecystectomy patients within a 3-month post-operative period. PROMs were identified as important considerations alongside more traditional clinical outcome measures, which were not independently associated with satisfaction in this cohort. Of the PROMs independently associated with higher post-cholecystectomy satisfaction, a return to normal leisure activities showed the strongest relationship, followed by the absence of wound pain, and high ratings of current health. This suggests that traditional clinical outcome measures fail to capture aspects important to satisfaction in cholecystectomy patients, and that the determination of PROMs can provide depth in understanding the post-operative recovery of these patients.

We reported a high overall satisfaction rate (96%) following cholecystectomy in respondents (with 71% reporting being “*very satisfied*”), which is comparable to rates ≥88% reported in other studies of cholecystectomy patients [10, 14–16]. In a high-volume centre such as ours [17], this surgical experience can have substantial benefits in surgical outcomes, particularly for high-risk patients. However, the association between PROMs and hospital volume requires further clarification, and so the clinical outcomes and PROMs reported here may not be generalisable to lower-volume hospitals. This high satisfaction may also be the result of volunteer bias from those with positive experiences [18], although the repeated attempts to contact patients aimed to minimise the impact this may have had [19]. Furthermore, respondents were found to be of a significantly higher age than non-respondents. This is concerning as older age has been found to correlate with higher post-surgical satisfaction [20, 21], hypothesised to be due to lower expectations and societal pressures [22]. This could have led to an overstated satisfaction rate, although studies on cholecystectomy patients have not found such an effect [23, 24].

We also identified a large difference in patient-reported and hospital-recorded 30-day complication rates (28.8 vs. 11.6%) which was significant [McNemar OR: 5.17, 95% CI (2.12–15.15), *p* < 0.001]. This is the first time that these complication rates have been compared using paired analysis in cholecystectomy patients, and our results agree with a previous study [25] which reported a similarly high discrepancy between surgeon and patient-reported complications in all type surgical patients. This warrants further investigation of the nature and outcome of these patient-reported complications and raises questions on whether complication rates evaluated only through hospital records adequately reflect the true complication rate experienced by patients. It would also be important to evaluate whether steps to ensure these patient-reported complications are appropriately addressed in the future. This could provide another potential avenue to improve post-surgery satisfaction in cholecystectomy patients.

The greatest strength of this study was the comprehensive and complete clinical information previously obtained for each patient. This allowed evaluation of the relationship between these surgical outcomes and subsequent PROMs and also highlights the flaws in the sole use of hospital records to investigate these outcomes. The patient-reported complications, such as wound infections, were far higher in comparison to hospital records. Therefore, surgeons may be inadvertently underestimating these risks when informing patients. In addition, the questionnaire used was a composite of three questionnaires previously utilised (two of which have been validated [8, 9]) and so this increases the comparability of the PROMs reported. In addition, the response rate (63%) to the questionnaire was higher than average for medical postal surveys [26], and this is similar to other such postal questionnaires performed within several months of cholecystectomy [27]. As response rates for postal questionnaires are often poor [28], we attempted to contact each participant before or after they received their questionnaire (with alternative telephone interviews) to minimise non-response and subsequent bias. However, this will likely still have impacted the results, given respondents were significantly older and had worse pre-operative health in terms of ASA classification.

There are several limitations in this study that should be considered. In addition to the potential biases already discussed, the questionnaire was conducted at a single point in time. As this was retrospective, it remains difficult to infer causality regarding associations identified. Furthermore, there was no standardisation regarding time to questionnaire completion between patients due to practical limitations of patient availability, and so respondents ranged from 1- to 3-month post-cholecystectomy. While this allowed comparison satisfaction between patients of different post-surgical periods, there was no prior or follow-up questionnaire, and so we cannot determine any progression in PROMs within the sample. As there can be substantial improvements in HRQoL within that time period [29], this could have had a confounding impact on the PROMs. However, this was not observed within our sample (Table 2), which could be expected within a routinely day-case procedure. Furthermore, few (15%) of patients reported recurrent symptoms compared to other studies reporting recurrence in up to 40% of patients [10, 30, 31]. However, as these studies involved longer periods of follow-up, patients may yet develop symptoms in the future. Finally, while the cohort was of a good size and the majority of patients reported positive experiences, this limited investigation of more negative outcomes. This was particularly evident in the small number of non-satisfied patients (*n* = 6), and so with a higher response rate, or an increased sample size, we could have improved statistical power.

Previous research into post-cholecystectomy satisfaction has predominantly focussed upon the impact of post-operative symptoms (such as pain [14, 15] or recurrent symptoms [10]), as opposed to the impact of cholecystectomy on patients’ daily lives. This is striking given the return to leisure activity showed the strongest independent association with satisfaction. Just one study has investigated the independent association between satisfaction and PROMs in this context [15], and those findings also support the absence of wound pain as being independently associated with positive opinions post-cholecystectomy.

The utility of PROMs to evaluate more complex surgical procedures remains undetermined; however, routine collection of post-operative PROMs data has already been implemented within the NHS in the context of hernia repair, hip and knee replacement, and varicose veins [4]. Therefore, there is a clear justification that assessment of post-cholecystectomy outcomes should also be supplemented by the use of PROMs in clinical practice order to facilitate further evaluation and improvement in patient care. With appropriate consideration of patient-specific factors, these could have a supportive role in informing treatment choice, and in monitoring patient recovery. In addition, while we cannot have confidence in inferences about causality in these associations, these factors nonetheless represent possible avenues to address in further improving the satisfaction rate in cholecystectomy patients. Although returning to leisure activity and opinions of current health are likely multifactorial in nature, patient-reported wound pain in particular represents a specific and feasible target for intervention. Further research could investigate whether steps to improve these factors could include: identifying and addressing barriers to the return to leisure activity in early post-cholecystectomy recovery, and ensuring adequate post-operative analgesia. The latter would need balanced against the side-effect profile of the analgesic regimen.

In conclusion, PROMs are the major determinants of patient satisfaction following cholecystectomy, with the return to leisure activity showing the strongest association. Therefore, when assessing outcomes following cholecystectomy, the measurement of clinical outcomes alone is inadequate and should be supplemented by the use of PROMs. Furthermore, this research also highlights a potential need for further steps to encourage return to normal activities and to ensure adequate post-operative analgesia to improve patient satisfaction.
